# *Rickettsia parkeri* and *Candidatus* Rickettsia andeanae in Gulf Coast Ticks, Mississippi, USA

**DOI:** 10.3201/eid1810.120250

**Published:** 2012-10

**Authors:** Flavia A.G. Ferrari, Jerome Goddard, Christopher D. Paddock, Andrea S. Varela-Stokes

**Affiliations:** Mississippi State University, Mississippi State, Mississippi, USA (F.A.G. Ferrari, J. Goddard, A.S. Varela-Stokes);; and Centers for Disease Control and Prevention, Atlanta, Georgia, USA (C.D. Paddock)

**Keywords:** Amblyomma maculatum, co-infection, Mississippi, spotted fever group rickettsiae, Rickettsia parkeri, Candidatus Rickettsia andeanae, bacteria, Gulf Coast ticks, Rickettsia, United States, ticks, vector-borne infections

**To the Editor:** *Rickettsia parkeri*, a spotted fever group *Rickettsia* (SFGR) bacterium, is transmitted by *Amblyomma maculatum*, the Gulf Coast tick (*1*). The prevalence of *R. parkeri* in Gulf Coast ticks has been reported as <42% in the United States, which is higher than reported rates of *R. rickettsii* (the cause of Rocky Mountain spotted fever) in *Dermacentor* species ticks. Misdiagnosis among SFGR infections is not uncommon, and *R. parkeri* rickettsiosis can cause symptoms similar to those for mild Rocky Mountain spotted fever (*1*). We evaluated infection rates of *R. parkeri* and *Candidatus* Rickettsia andeanae, a recently identified but incompletely characterized SFGR, in Gulf Coast ticks in Mississippi, USA.

During May–September 2008–2010, we collected adult Gulf Coast ticks from vegetation at 10 sites in Mississippi. We extracted genomic DNA from the ticks using the illustra tissue and cells genomicPrep Mini Spin Kit (GE Healthcare Life Sciences, Piscataway, NJ, USA). We tested amplifiable tick DNA by PCR of the tick mitochondrial 16S rRNA gene (*2*). We tested for molecular evidence of any SFGR species by nested PCR of *rompA* (rickettsial outer membrane protein A gene) (*1*). Samples positive for SFGR were subsequently tested by using species-specific *rompA* PCR for *R. parkeri* (*3*) and *Candidatus* R. andeanae (*4*). All PCRs included 1) a positive control of DNA from cultured *R. parkeri*– (Tate’s Hell strain) or *Candidatus* R. andeanae–infected Gulf Coast ticks and 2) a negative control of water (nontemplate). PCR products were purified by using Montage PCR Centrifugal Filter Devices (Millipore, Bedford, MA, USA) and sequenced by using Eurofins MWG Operon (Huntsville, AL, USA). We generated consensus sequences using ClustalW2 (http://www.ebi.ac.uk/Tools/msa/clustalw2/) alignment and identified the sequences using GenBank BLAST searches (www.ebi.ac.uk/Tools/clustalw2/).

Proportions of ticks infected with SFGR, by region and year, were compared separately by using Fisher exact test followed by pairwise comparisons with a Bonferroni adjustment (PROC FREQ, SAS for Windows, V9.2; SAS Institute, Cary, NC, USA). For all analyses, p<0.05 was considered significant. An index of co-infection was calculated by using the formula IC = ([O – E]/N) × 100, in which IC is index of co-infection, O is number of co-infections, E is expected occurrence of co-infection caused by chance alone, and N is total number of ticks infected by either or both *Rickettsia* species. A χ^2^ test was used to determine statistical significance (*5*).

A total of 707 adult Gulf Coast ticks were collected during the 3 years (350 in 2008, 194 in 2009, and 163 in 2010). Tick mitochondrial 16S rRNA gene was detected in 698 (98.7%), of which 128 (18.3%) were positive for SFGR DNA, comprising 106 (15.2%) positive only for *R. parkeri*, 10 (1.4%) positive only for *Candidatus* R. andeanae, and 12 (1.7%) co-infected with *R. parkeri* and *Candidatus* R. andeanae (Table). Positive test results from 22 ticks singly or co-infected with *Candidatus* R. andeanae were confirmed by sequencing.

**Table:** PCR results for adult *Rickettsia parkeri*– and *Candidatus* Rickettsia andeanae–infected Gulf Coast ticks (*Amblyomma maculatum*) collected from 10 sites in Mississippi, USA, 2008–2010*

Location (no. collection sites)	No. ticks	No. (%; 95% CI) SFG *rompA*	No. (%; 95% CI) *R. parkeri* only	No. (%; 95% CI) *Candidatus* R. andeanae only	Expected no. (%) co-infected ticks	No. (%; 95% CI) co-infected ticks
North (4)	257	49 (19.1; 14.5–24.4)†	48 (18.7; 14.1–24)‡	0§	0.19 (0.07)	1 (0.4; 0–2.1)¶
Central (1)	38	4 (10.5; NA)	1 (2.6; NA)	2 (5.3; NA)	0.16 (0.42)	1 (2.6; NA)
South (5)	403	75 (18.6; 14.9–22.8)†	57 (14.1; 10.9–17.9)‡	8 (2.0; 0.9–3.9)§	2.99 (0.74)	10 (2.5; 1.2–4.5)¶
Total (10)	698	128 (18.3; NA)	106 (15.2; NA)	10 (1.4; NA)	3.65 (0.52)	12 (1.7; NA)

*The estimated value of co-infection caused by chance alone (E) was calculated by using the formula E = (a + b)(a + c) / (a + b + c + d) (*5*), where a = no. ticks infected with both *Rickettsia* species, b = no. ticks infected only with *R. parkeri*, c = no. ticks infected only with *Candidatus* R. andeanae, and d = no. ticks not infected with either *Rickettsia* species. SFG *rompA*, spotted fever group rickettsial outer membrane protein A gene. NA, not applicable. †p = 0.9187. ‡p = 0.1275. §p = 0.0257. ¶p = 0.0578 (comparison of prevalence from northern and southern sites only).

Most (94.6%) ticks were from northern (n = 260) and southern (n = 409) Mississippi (Technical Appendix Figure). No significant difference in the number of *R. parkeri*–infected ticks between northern and southern Mississippi was observed (p = 0.13) (Table). However, significantly more ticks were singly infected with *Candidatus* R. andeanae in southern sites than in northern sites (p = 0.03). The infection rate for co-infected ticks in southern sites was higher than that in northern sites (p = 0.06). Among the 3 collection years for northern and southern sites, only the prevalence of *R. parkeri* in singly infected ticks differed significantly (p = 0.01) (data not shown); the infection rate was significantly greater during 2010 than during 2009 (p = 0.003, α/3 = 0.02). The overall index of co-infection with *R. parkeri* and *Candidatus* R. andeanae was 6.5, statistically higher than expected by chance alone (Table) (p<0.0001).

The overall prevalence of infection with SFGR species in Gulf Coast ticks sampled was 18.3%; 15.2% of ticks were singly infected with *R. parkeri*, and 1.7% were infected with *R. parkeri* and *Candidatus* R. andeanae. As reported, the frequency of *R. parkeri* in Gulf Coast ticks is generally high, ranging from ≈10% to 40% (*3*,*4*,*6*–*8*). We found approximately 1 *R. parkeri*-infected Gulf Coast tick for every 6 ticks tested, suggesting that infected Gulf Coast ticks are commonly encountered in Mississippi. Because Gulf Coast ticks are among the most common human-biting ticks in Mississippi (*9*), awareness of *R. parkeri* rickettsiosis should be increased in this state. We identified *Candidatus* R. andeanae in ≈3% of Gulf Coast ticks in Mississippi; this frequency is similar to those reported in other studies of Gulf Coast ticks in the southern United States (*4*,*6*). Our finding of co-infected Gulf Coast ticks is at a frequency significantly higher than expected from chance alone. The biologic role of co-infections of Gulf Coast ticks with *R. parkeri* and *Candidatus* R. andeanae remains to be determined.

Technical Appendix Distribution of Gulf Coast ticks and cases of rickettsiosis.
