# Supplementary material for: Rickettsia parkeri and Candidatus Rickettsia andeanae in Gulf Coast Ticks, Mississippi, USA
Source: Emerg Infect Dis. 2012 Oct;18(10):1705–7. doi: 10.3201/eid1810.120250 (PMC3471625; doi:10.3201/eid1810.120250)
Supplement: Technical Appendix — Distribution of Gulf Coast ticks and cases of rickettsiosis. [file 12-0250-Techapp-s1.pdf]

Article DOI: http://dx.doi.org/10.3201/eid1810.120250

Rickettsia parkeri and *Candidatus* Rickettsia andeanae in Gulf Coast Ticks, Mississippi, USA

Technical Appendix

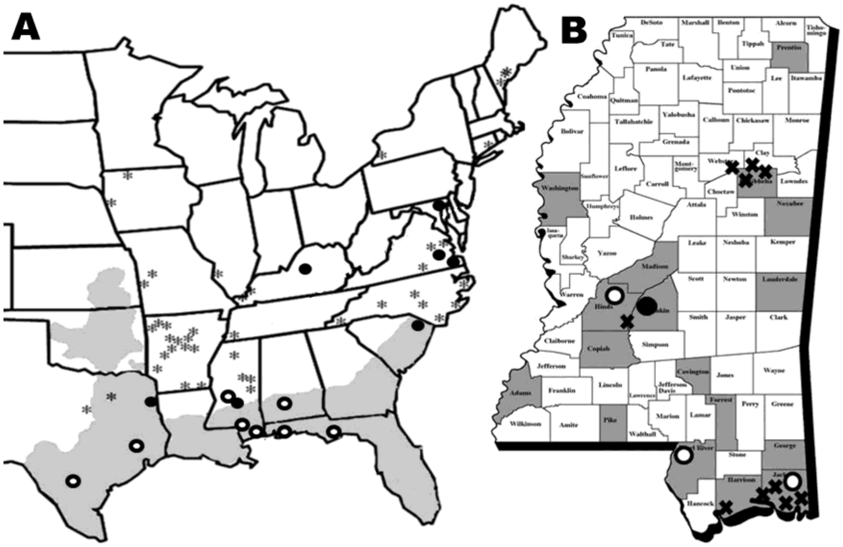

Technical Appendix Figure. Distribution of Gulf Coast ticks and cases of rickettsiosis. Solid circles indicate confirmed cases of *Rickettsia parkeri* rickettsiosis, and open circles indicate probable cases of *R. parkeri* rickettsiosis noted by Paddock et al. (*1*) and Cragun et al. (*2*). A) Gray area indicates the most established distribution of Gulf Coast ticks (*Amblyomma maculatum*); asterisks (*) indicate documented collection of these ticks in incidental reports (may or may not reflect permanent populations). B) Gray shading indicates Mississippi counties where Goddard and Paddock reported the occurrence of Gulf Coast ticks and locations of previously reported cases of *R. parkeri* rickettsiosis in Mississippi (*3*). X indicates tick collection sites for this study. Map adapted with permission from Teel et al. (*4*).

References

1. Paddock CD, Finley RW, Wright CS, Robinson HN, Schrodt BJ, Lane CC, et al. *Rickettsia parkeri* rickettsiosis and its clinical distinction from Rocky Mountain spotted fever. Clin Infect Dis. 2008;47:1188–96. PubMed http://dx.doi.org/10.1086/592254

2. Cragun WC, Bartlett BL, Ellis MW, Hoover AZ, Tyring SK, Mendoza N, et al. The expanding spectrum of eschar-associated rickettsioses in the United States. Arch Dermatol. 2010;146:641–8. PubMed http://dx.doi.org/10.1001/archdermatol.2010.48

3. Goddard J, Paddock CD. Observations on distribution and seasonal activity of the Gulf Coast tick in Mississippi. J Med Entomol. 2005;42:176–9. PubMed http://dx.doi.org/10.1603/0022-2585(2005)042[0176:OODASA]2.0.CO;2

4. Teel PD, Ketchum HR, Mock DE, Wright RE, Strey OF. The Gulf Coast tick: a review of the life history, ecology, distribution, and emergence as an arthropod of medical and veterinary importance. J Med Entomol. 2010;47:707–22. PubMed http://dx.doi.org/10.1603/ME10029
